# New Entries in the Lottery of Facial GWAS Discovery

**DOI:** 10.1371/journal.pgen.1006250

**Published:** 2016-08-25

**Authors:** Peter Claes, Mark D. Shriver

**Affiliations:** 1 Department of Electrical Engineering (ESAT/PSI), KU Leuven, Leuven, Belgium; 2 Medical Imaging Research Center, UZ Leuven, Leuven, Belgium; 3 Department of Anthropology, Penn State University, University Park, Pennsylvania, United States of America; Stanford University School of Medicine, UNITED STATES

We are all familiar with the strong genetic control of faces seen in the almost indistinguishable facial appearance of identical twins, similarities within families and populations, as well as shared facial characteristics in medical syndromes. Nevertheless, our understanding of the genetic architecture of normal-range human facial variation has remained largely uninvestigated, until recently.

**Fig 1:**
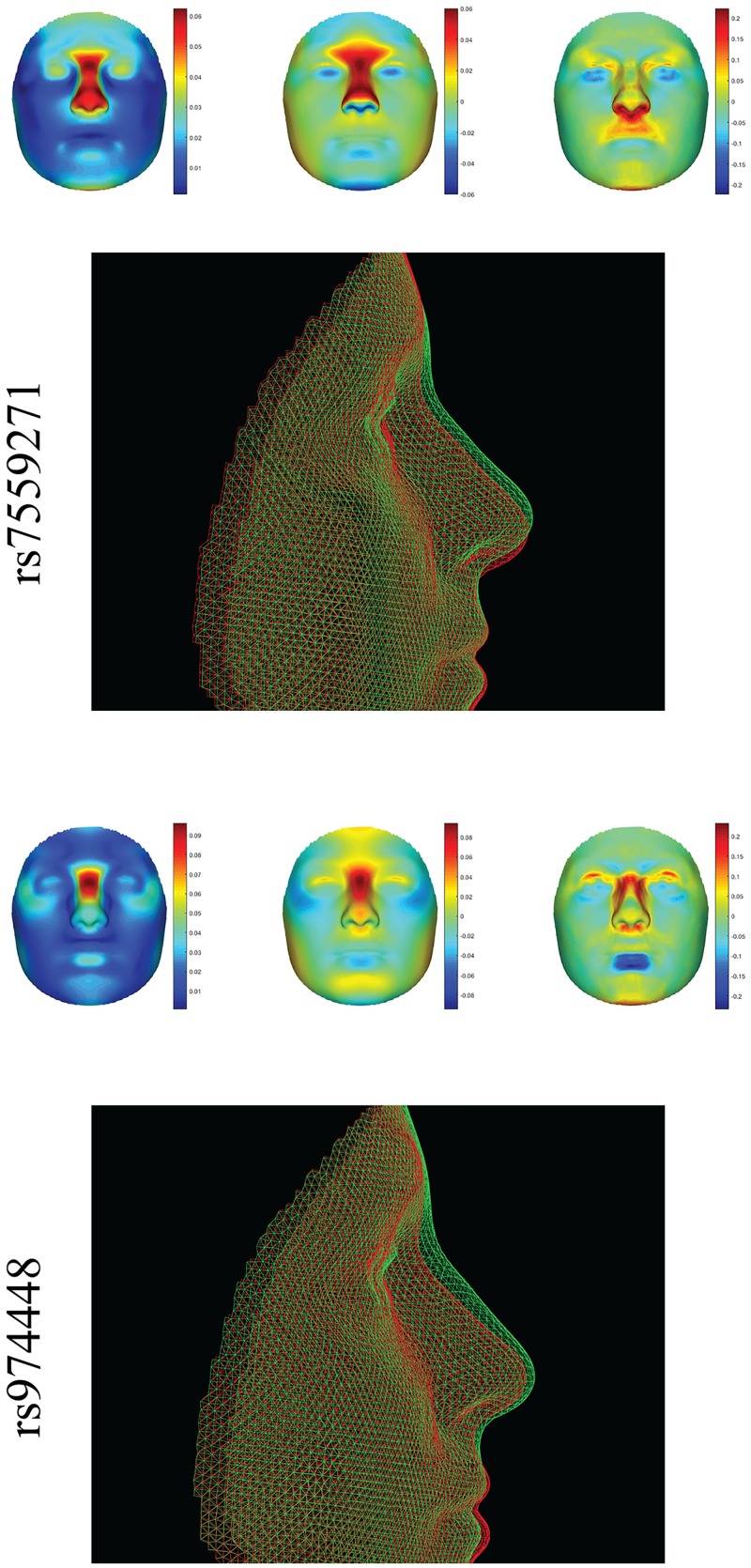
Full facial effects of two different loci in *PAX3*, rs7559271 (top) [5], and rs974448 (bottom) [4]. Both loci were tested for replication in [2] with a *p*-value of 0.392 and 0.002, respectively. Courtesy of the University of Pittsburgh, the Pittsburgh data sample [2] was processed with spatially-dense geometric morphometric techniques as outlined in [7] to expose the effects of both SNP variations onto the full facial surface. Color plots from left to right, the effect magnitude (red: strong effect, blue: no effect), the normal displacement (red: minor allele phenotype [mAP], in comparison to major allele phenotype [MAP], is more prominent, blue: mAP is less prominent, green: no difference in prominence), and area changes (red: mAP displays a larger area, blue: mAP display a smaller area, green: no difference in area). Overlays: mAP/MAP are visualized as green/red wireframes. Both SNP effects are clearly focused on the nose, with the common aspect of nose ridge and bridge elevation. In contrast to rs7559271, rs974448 also influences the relative position of the nose to the eyes and lower orbits. Phenotypic distances, such as nasion to orbit used in [4] and Intercanthal width used in [2], properly capture these relative changes, and therefore, obtained significant results with rs974448. The same phenotypic distances are not representative for the effects of rs7559271, for which replication in [2] failed. Generating complete descriptions of how factors like particular SNPs affect facial variation, as shown here, can facilitate the proper definition of phenotypic measurements to be used in future replication efforts.

Cole et al., 2016 [1], and Shaffer et al., 2016 [2], present us with additional insights into the complex puzzle that is facial genetics. These two studies along with one other, Ahidkari et al., 2016 [3], represent the second round of genome-wide association studies (GWAS) on facial morphology; the first round appeared in 2012 [4,5]. Although genes affecting the development of the face have been studied for decades using pedigree-based analyses, molecular biology, and animal models, the discovery of genetic variants affecting normal-range facial variation using GWAS is still in its infancy. The progression of facial GWAS has been impeded by several factors, including a lack of appropriate 3-D facial scanning systems, the significant efforts required to collect participants, subsequent rigorous statistical analyses, and the lack of methods for functional follow-up analyses [6]. The two facial GWAS from 2012 provided a handful of associated genes, one of which, *PAX3*, was significant in both studies (Fig 1). Evidence confirming a role for *PAX3* is presented in the Shaffer et al. report, which also replicated three other genes from the 2012 GWAS: *CACNA2D3*, *C5orf50*, and *PRDM16* [2]. The failure to replicate the other associations could result from many factors in study design, such as consistent facial phenotyping as noted by [2] and population background as noted by [1]. Future studies bear the responsibility to investigate earlier findings for replication, and with these two new facial GWAS, this list has tripled in length.

These two GWAS provide some new and exciting loci, datasets, and approaches. The increased list of craniofacial genes brings us closer to a better understanding of facial development and disease. They also increase our optimism in realizing unresolved medical and forensic applications that have compelling social implications. Although many genetic conditions that involve clinically significant (atypical) patterns of facial development have been mapped, and these studies have arguably established most of what we know about facial genetics, many individual cases remain undiagnosed. A better understanding of the genetics of normal-range craniofacial development can and should help in the delineation of which genes underlie these conditions. Generally, this process has been inverted, with information on the genetic determinants of disorders serving to help identify candidate genes for investigations into normal-range facial variation [7]. Understanding the genetic architecture of normal-range facial variation would also assist efforts to predict faces from DNA [8], a forensic application that has generated much public interest.

One major strength of the two new studies is the availability of the facial shape and genotype data through FaceBase (www.facebase.org) and dbGaP (www.ncbi.nlm.nih.gov/gap), respectively. Both studies also provide examples for how to deal with imperfections and confounders typically encountered in 3-D facial datasets. For example, they deal with different scanning equipment, facial imaging and phenotyping quality control, and the issue of manual versus automated landmarking. Shaffer et al. [2] used inter-landmark distances that are known to be clinically relevant indicators. In contrast, Cole et al. [1] used both inter-landmark distances and geometric morphometrics, an approach to shape analysis that has been used extensively in evolutionary and developmental biology. Alternatively, Adhikari et al. [3] used an observer-based facial phenotyping approach where faces are subjectively assessed by human observers either into classes or on an ordinal scale based on the presence or absence of certain characteristics. Each phenotyping approach has its own advantages, but it is challenging to relate different kinds of facial measurements. More complete descriptions of how factors like particular SNPs affect facial variation, as displayed in Fig 1, will provide a stronger basis for making comparisons between the effects uncovered in one association study to those found in the next GWAS as well as comparisons between normal-range effects and the effects modeled in craniofacial disease.

The two new studies are ambitious in recruiting participants whose ages span from young [1] to a wide range [2]. Facial growth and development and, later in life, facial aging are both complex and non-linear phenomena. Therefore, associations found in children may not be found in adults and vice versa. However, quoting Cole et al. [1] in their response to reviewers, “…that does not negate the relevance of studying facial shape in children, in which loci that contribute to developmental aspects of facial shape may be more engaged and relevant in children (with still-developing faces) than in adults.” Furthermore, in their response to reviewers, Shaffer et al. [2] said, “Failure to completely account for the effects of age variation on traits is highly unlikely to result in false positive genome-wide signals. That is, age cannot act as an epidemiological confounder because age does not alter the constitutional genome…” Also of note is the use of non-European populations, such as a cohort of East African children [1], and an admixed Latin-American sample [3].

In moving forward, it is worthwhile to reflect briefly on a somewhat distracting obsession in GWAS, namely, achieving genome-wide statistical significance. In a European cohort, the significance threshold is 5*10^-8, while in an African cohort, due to shorter haplotype blocks, an even more stringent threshold (2.5*10^-8) is used [9]. The current trend in GWAS is to increase the number of SNPs (up to 10M) throughout the genome, increasing the odds of including relevant SNPs, which is made possible thanks to increased ease of genotyping and genotype imputation. However, this is not the only way forward. Both of these new facial GWAS illustrate the use of contemporary genetic methodology such as meta-analyses and linear mixed models. In association studies, linear mixed models are emerging as a method of choice [10] where recent promising advancements have been made [11]. However, in striking contrast, facial phenotypes are still reduced to a limited series of a priori simplifications that are analyzed individually. However, the solution is not simply to increase the number of facial measurements, increasing the odds of including relevant phenotypic traits. The computational burden and, more importantly, the considerations of statistical power make this approach inefficient.

An alternative approach involves identifying phenotypic measurements taken from the same facial subunit or module and using these measures jointly in multivariate association analyses. The human body is organized into multiple organ systems, each of which is oriented towards a specific task in accordance to its function, anatomy, and embryological origin. Facial shape, likewise, represents a complex system that is experimentally and developmentally separable into several modules—for instance, bone and muscle cells for structural support and movement; skin cells for transpiration, protection, and temperature regulation; and retinal cells for image processing. Combining linear measurements, e.g., using factor analysis, can facilitate the description of unmeasured facial traits. Simply measuring and combining two sides of a triangle also captures information on the unmeasured third side. There is no need to also measure and investigate the third side of the triangle separately. The primary challenge lies in defining relevant groupings of measurements. Combining either too many or unrelated measurements in a multivariate analysis will reduce statistical power. Thus, defining which measures are related is not trivial. Further research on facial anatomy and phenotypic correlation, heritability and genetic correlation, modularity and integration, as well as adaptation and evolution will provide additional clues for grouping facial traits. The work and data presented in these new facial GWAS already allow testing of such an approach.
